# Burden of Healthcare-Associated Infections and Antimicrobial Resistance in a Romanian Cardiovascular and Transplant Center: Factors Associated with Mortality

**DOI:** 10.3390/antibiotics14090926

**Published:** 2025-09-13

**Authors:** Mihaela-Alexandra Budianu, Cristina Nicoleta Ciurea, Liviu Moraru, Septimiu Voidăzan

**Affiliations:** 1 Department of Epidemiology, University of Medicine, Pharmacy, Science and Technology “George Emil Palade” of Tîrgu Mureș, 540139 Târgu Mureș, Romania; 2 Department of Microbiology, University of Medicine, Pharmacy, Science and Technology “George Emil Palade” of Tîrgu Mureș, 540139 Târgu Mureș, Romania; 3 Department of Anatomy, University of Medicine, Pharmacy, Science and Technology “George Emil Palade” of Tîrgu Mureș, 540139 Târgu Mureș, Romania

**Keywords:** HAIs, ICU, antibiotic resistance, *Klebsiella pneumoniae*, *Acinetobacter baumannii*

## Abstract

Background/Objectives: Healthcare-associated infections (HAIs) are a major burden in tertiary hospitals, particularly in high-risk populations such as cardiovascular and transplant patients. The emergence of antimicrobial resistance (AMR) further complicates management, contributes to poor outcomes and increases costs. This study aimed to describe the burden of HAIs, the antimicrobial resistance patterns of the main pathogens, and to identify predictors of mortality among patients hospitalized in a Romanian cardiovascular and transplant center. Methods: We conducted a retrospective study including all patients with HAIs reported to the Infection Prevention and Control Service of the Emergency Institute for Cardiovascular Diseases and Transplantation, Târgu Mureș, Romania, between 2023 and the first quarter of 2025. Descriptive statistics summarized demographic and clinical data. Univariable and multivariable logistic regression were used to assess risk factors for mortality. Antimicrobial resistance was evaluated through cumulative antibiograms. Results: A total of 139 HAIs were reported during the study period, with a prevalence of 1.05% in 2023 and 1.0% in 2024. The most common pathogens were SARS-CoV-2 (15%), *Klebsiella pneumoniae* (14%), and *Acinetobacter baumannii* (13%). Overall, all-cause in-hospital mortality was 32%, with 30% of deaths associated with *A. baumannii* and 27% with *K. pneumoniae*. More than half of bacterial isolates were resistant to multiple antibiotic classes; 22% were extensively drug-resistant. ICU admission and treatment with more than three antibiotics were independently associated with mortality. Conclusions: Although the prevalence of HAIs in our study was low, the cases that occurred in cardiovascular and transplant patients were often severe, frequently involved multidrug-resistant organisms, and were associated with high mortality. Strengthening infection prevention and antimicrobial stewardship is essential to reduce the impact of HAIs in this vulnerable population.

## 1. Introduction

Healthcare-associated infections (HAIs) are a major global public health challenge, contributing significantly to patient morbidity, prolonged hospitalization, increased costs, and mortality [1,2,3,4,5]. Although their incidence cannot be reduced to zero, effective infection prevention and control (IPC) measures can substantially lower their burden [6,7,8,9,10,11]. Within the European Union, an estimated 3.5 million patients develop HAIs annually, leading to more than 90,000 deaths [12,13,14,15,16].

Antimicrobial resistance (AMR) and HAIs can also be viewed through broader frameworks that help explain how they arise and spread. The epidemiological triad represented by: agent, host, and environment offers one such perspective, resistant bacteria are the agents, patients provide the hosts, and hospitals represent the environment where these infections take hold and multiply [17]. Looking at HAIs in this way shows that they are not isolated clinical events, but the result of constant interaction between microbes, people, and the settings where they are treated.

In Romania, the official reported prevalence of HAIs is around 1%, far below the European average of 7% [13,18,19,20]. This discrepancy reflects underreporting rather than a lower incidence, with systemic deficiencies in surveillance, insufficient infection control staff, and fear of administrative sanctions contributing to incomplete data reports [21]. ECDC estimates suggest a true prevalence closer to 2.6% [13], while a national survey reported 4.1% overall, reaching 14.9% in intensive care units [19]. The most frequently identified pathogens include resistant *Enterobacterales* (Extended Spectrum Beta-Lactamase-producing *Enterobacterales*, Carbapenem-Resistant *Enterobacterales*), *Clostridioides difficile*, and methicillin-resistant *Staphylococcus aureus* [20,22].

High antibiotic consumption further increased AMR [23,24]. Romania consistently ranks among the EU countries with the highest outpatient use of (>25 DDD/1000/day), largely broad-spectrum penicillin, cephalosporins, and fluoroquinolones [13,25,26]. In hospitals, use exceeds 70 DDD/1000 patient-days, dominated by penicillin–beta–lactamase inhibitor combinations and carbapenems [21]. Self-medication, over-the-counter access, and limited implementation of stewardship programs exacerbate inappropriate prescribing, creating problems in hospitals, as well as in the community [24,26,27,28].

These factors place Romanian patients, particularly those in intensive care and transplant centers, at high risk for multidrug-resistant infections and poor outcomes, such as increased morbidity and mortality. The aim of this study was to evaluate the burden of HAIs in a Romanian cardiovascular and transplant center, describe the antimicrobial resistance patterns of the most common pathogens, and identify factors associated with mortality among high-risk patients (children with congenital heart defects, recipients of heart transplants, or with cardiovascular diseases).

## 2. Results

### 2.1. Sample Characteristics and Microorganisms

During the study period, 139 cases were reported as HAIs, most of which occurred in 2023. In 2023, the prevalence of healthcare-associated infections (HAIs) in the institute was 1.05% (63/5971 admissions), compared with 1.0% in 2024 (58/6329 admissions). Overall, 58% of patients with HAIs were male, 36% were younger than 18 years (including 30% under 4 years), and 38% were older than 65 years. The median time from admission to HAI detection was 12 days (IQR: 7–24). Half of all cases occurred in intensive care units (ICUs), and among patients with bacterial infections, two-thirds were ICU-related, with a percentage of 53% involving antimicrobial-resistant organisms.

**Figure 1:**
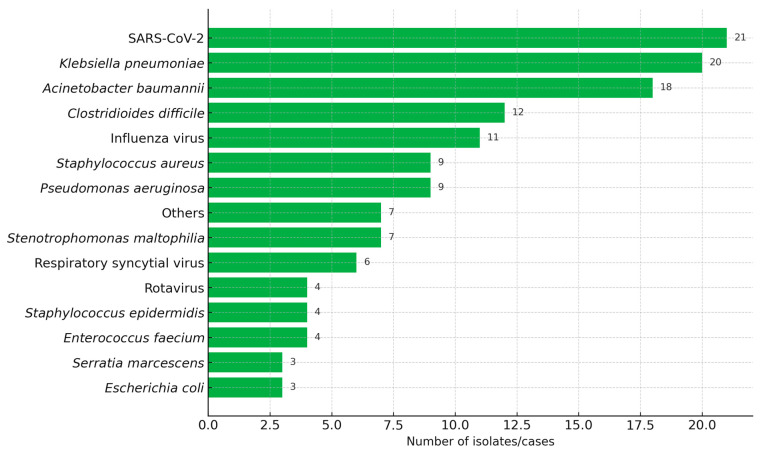
Distribution of microorganisms causing healthcare-associated infections (HAIs).

The most frequently identified pathogens were SARS-CoV-2 (15%), *K. pneumoniae* (14%), and *A. baumannii* (13%) (Figure 1). Overall, respiratory tract infections were the most common with 78 cases (56%), followed by sepsis with 36 (26%), and digestive tract infections 16 (12%).

In the ICU, *A. baumannii* was the most common pathogen implicated in HAIs (n = 18; 26%), followed by *K. pneumoniae* (n = 16; 23%), and *Pseudomonas aeruginosa* (n = 7; 10%).

The overall all-cause in-hospital mortality during our study period was 32% (44/139). Among fatal cases, 30% were associated with *A. baumannii* and 27% with *K. pneumoniae.* Mortality was significantly associated with ICU admission (*p* < 0.001), respiratory tract infection (*p* < 0.001), and infection with resistant pathogens (*p* = 0.02); mortality among patients with an *A. baumannii* infection was 72%.

The median number of antibiotics received was 4 (IQR: 3–5). Most patients received 3–5 types of antibiotics, but a small number required ≥7, indicating a right-skewed distribution driven by resistant infections. Antibiotic exposure was high, with 44% of patients receiving 2–3 agents and 55% more than 3; increased antibiotic usage correlated significantly with resistance (*p* = 0.001).

### 2.2. Antimicrobial Resistance

During the study period, 96 patients were diagnosed with bacterial HAIs, with a case-fatality rate of 43% (41/96).

More than half of those cases involved microorganisms with antimicrobial resistance, with a total of 51 resistant phenotypes identified during the study period. These included 11 ESBL-producing *Enterobacterales*, 9 carbapenemase-producing *Enterobacterales* (CPE), 1 multidrug-resistant (MDR) *P. aeruginosa*, 5 methicillin-resistant *S. aureus* (MRSA), 4 vancomycin-resistant Enterococcus (VRE), and 21 extensively drug-resistant (XDR) Gram-negative bacilli.

**Figure 2:**
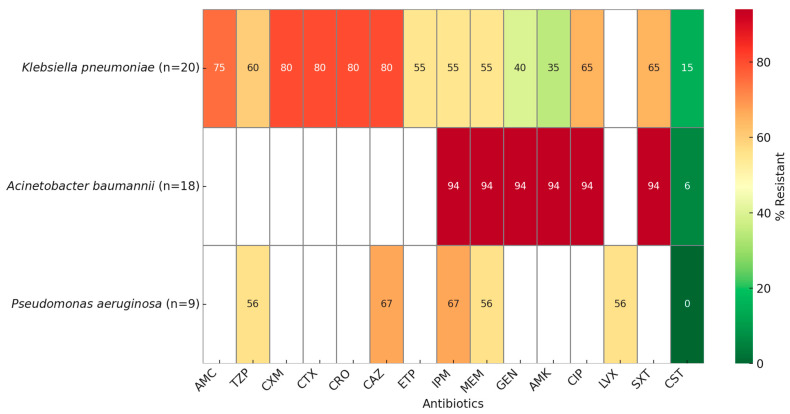
Cumulative antibiogram of Gram-negative isolates (AMC—Amoxicillin–clavulanic acid; TZP—Piperacillin–tazobactam; CXM—Cefuroxime; CTX—Cefotaxime; CRO—Ceftriaxone; CAZ—Ceftazidime; ETP—Ertapenem; IPM—Imipenem; MEM—Meropenem; GEN—Gentamicin; AMK—Amikacin; CIP—Ciprofloxacin; LVX—Levofloxacin; SXT—Trimethoprim–sulfamethoxazole; CST—Colistin). Resistance rates are expressed as percentages of isolates tested against each antimicrobial agent. White cells indicate antibiotics not tested.

The cumulative antibiogram showed high resistance rates among Gram-negative pathogens isolated from healthcare-associated infections. *K. pneumoniae* showed resistance rates of 75–80% to most cephalosporins (Cefuroxime, Ceftriaxone, Cefotaxime, Ceftazidime), fluoroquinolones (Ciprofloxacin 65% and β-lactam/β-lactamase inhibitor combinations) (Piperacillin–tazobactam 65%). Resistance to aminoglycosides was lower (Amikacin 35%, Gentamicin 40%), while carbapenem resistance was 55%. Colistin retained the highest activity, with only 15% resistance (Figure 2).

*A. baumannii* exhibited a critical resistance profile, with resistance rates of 94% across aminoglycosides, fluoroquinolones, carbapenems, and Piperacillin–tazobactam. Colistin remained the most effective agent, although resistance was still observed in 6% of isolates.

*P. aeruginosa* isolates showed moderate to high resistance, ranging from 56% (Amikacin, Ciprofloxacin, Meropenem, Piperacillin–tazobactam) to 67% (Ceftazidime, Imipenem). All isolates were susceptible to Colistin (0% resistance).

**Figure 3:**
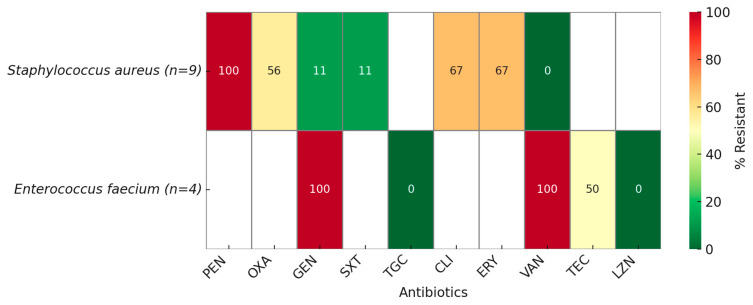
Cumulative antibiogram of gram-positive isolates (PEN—Penicillin; OXA—Oxacillin; GEN—Gentamicin; SXT—Trimethoprim–Sulfamethoxazole; TGC—Tigecycline; CLI—Clindamycin; ERY—Erythromycin; VAN—Vancomycin; TEC—Teicoplanin; LZD—Linezolid). Resistance rates are expressed as percentages of isolates tested against each antimicrobial agent. White cells indicate antibiotics not tested.

Among *S. aureus* isolates, penicillin resistance was universal and 56% were oxacillin resistant (MRSA). High resistance was also observed for Clindamycin and Erythromycin (67%), most isolates remained susceptible to Trimethoprim–sulfamethoxazole and Gentamicin, and all were susceptible to Vancomycin (Figure 3).

*Enterococcus faecium* isolates exhibited 100% resistance to Gentamicin, and all were Vancomycin resistant (VRE). Resistance to Teicoplanin was detected in 50% of isolates, while Linezolid retained full activity.

### 2.3. Univariable Analysis of Factors Associated with Death Among Patients with Bacterial Infections

In univariable analysis, deceased patients were less likely to be minors compared with those older than 65 years (OR = 0.28; 95% CI: 0.10–0.76; *p* = 0.01) (Table 1).

Patients who died were 9.6 times more likely to have been admitted to the ICU at the time of HAI diagnosis (95% CI: 3.00–30.57; *p* < 0.001) and were more likely to have an AMR pathogen (OR = 4.41; 95% CI: 1.83–10.63; *p* = 0.001). When evaluating the association between resistance phenotypes and mortality, infections caused by carbapenemase-producing *Enterobacterales* (CPE) (OR = 3.70; 95% CI: 1.95–59.94; *p* = 0.006) and extensively drug-resistant (XDR) organisms (OR = 7.72; 95% CI: 2.40–24.78; *p* = 0.001) were significantly associated with increased risk of death.

Compared with deceased patients who received 1–3 antibiotic classes, those who died were 11.7 times more likely to have received >3 antibiotics (OR = 11.99; 95% CI: 4.26–33.69; *p* < 0.001).

Lower mortality was observed among patients admitted in 2024 or early 2025, although this association was not statistically significant.

### 2.4. Multivariable Analysis of Factors Associated with Death Among Patients with Bacterial Infections

In the multivariable analysis, mortality was independently associated with ICU admission at the time of HAI diagnosis (OR=5.89; 95% CI: 1.39–24.91; *p* = 0.016) and with receipt of more than three antibiotic classes compared with three or fewer (OR 6.79; 95% CI 2.00–23.03; *p* = 0.002) (Table 2).

Confirmed AMR infection showed a positive but non-significant association with death (OR 1.52; 95% CI 0.47–4.84; *p* = 0.47).

## 3. Discussion

HAIs remain a serious public health concern, contributing substantially to increased morbidity and mortality, with higher healthcare costs [29]. In our study, most bacterial infections were identified in intensive care units, reflecting international observations that ICU patients are particularly vulnerable because of invasive procedures, frequent exposure to broad-spectrum antibiotics, and compromised immune status [22,30]. We also found that children younger than 4 years and adults over 65 years together accounted for nearly 70% of all cases, confirming the well-recognized susceptibility of patients at the extremes of age to severe outcomes [31,32,33,34].

One of the most important findings of this study was the high proportion of infections caused by resistant organisms (53%), compared with 47% due to susceptible bacteria. Gram-negative species were dominant, especially *K. pneumoniae* and *A. baumannii*. Mortality was significantly higher in patients with infections with resistant bacteria, with almost three-quarters of them dying compared to only 27% of those infected with susceptible strains. The strongest associations with a poor outcome were observed in infections caused by *K. pneumoniae*, *A. baumannii*, and *P. aeruginosa*. These results are consistent with reports from tertiary hospitals in China and other regions, as well as with other studies that described similar mortality patterns in multidrug-resistant Gram-negative and MRSA infections [4,35,36,37]. The resistance rates we observed of 94% for *A. baumannii* and 80% for *K. pneumoniae* highlight the global concerns regarding the spread of multidrug-resistant organisms [38,39,40].

*K. pneumoniae*, *A. baumannii*, and *P. aeruginosa*, the most commonly reported bacteria in our study, were previously acknowledged by the World Health Organization as serious threats. In 2017, carbapenem-resistant *A. baumannii* and *P. aeruginosa*, along with resistant Enterobacteriaceae (including *K. pneumoniae*), were placed at the highest priority level [41]. The updated list (2024) maintains their position [42]. While our findings emerge from a defined local setting, their alignment with these international designations gives these findings wider significance. Our results therefore speak not only to local patterns of resistance, but also to a global challenge that demands coordinated action and ongoing surveillance of antimicrobial resistance worldwide.

High antibiotic usage was another notable finding, with more than half of patients (55%) receiving three or more antibiotic classes. Patients with resistant infections required treatment with more antibiotic classes (often 4–5), reflecting the challenges of managing resistance and contributing to significantly higher costs. This pattern has been repeatedly reported in the literature and highlights the urgent need to strengthen antibiotic stewardship programs [24,27]. Univariable analysis also pointed to advanced age, ICU admission, multidrug-resistant infections, and extensive antibiotic use as key risk factors for mortality, findings that are in line with the other published studies [34,43].

Respiratory tract infections emerged as the most frequent type of HAI, accounting for over half of all cases, followed by sepsis and digestive tract infections, the majority being caused by *C. difficile*. Mortality associated with respiratory infections reached 73%, clearly linking infection site to prognosis [32,44,45]. These infections are frequently related to intubation and mechanical ventilation. Two measures are considered essential to prevent ventilator-associated pneumonia: regular monitoring of endotracheal tube cuff pressure—since insufficient pressure allows leakage of contaminated secretions while excessive pressure can cause ischemic injury—and consistent oral hygiene. The WHO recommends oral care every four hours with single-use kits, including cleaning of the teeth, tongue, and palate. In Romania, however, the implementation of these measures is limited by staff shortages and lack of adequate resources, creating significant barriers to prevention.

Our findings are consistent with national data showing that more than half of HAIs in Romanian hospitals originate in ICUs [18]. While factors such as prolonged hospitalization, invasive procedures, and intensive antibiotic exposure explain much of this risk, adherence to infection prevention and control protocols could still lower infection rates in these settings.

In our study, 139 HAI cases were identified through active surveillance, corresponding to a prevalence of approximately 1%, similar to official Romanian reports. We therefore do not interpret our findings as evidence of underreporting within our hospital, but rather as confirmation that reported prevalence in Romania remains much lower than the European average of 7% [13]. This discrepancy likely reflects differences in surveillance methodology, case ascertainment, and reporting practices at the national level, rather than a genuinely lower incidence of HAIs.

Poor compliance with national IPC guidelines and persistent underreporting of HAIs, previously documented in Romanian studies [19], remain major challenges. Strengthening surveillance systems, integrating cumulative antibiogram data into treatment decisions, and encouraging non-punitive reporting to Health Department would be important steps forward. Even relatively simple interventions, such as auditing hand hygiene, improving disinfection training, and closely monitoring sterilization and disinfection, could significantly reduce HAI incidence and mortality associated with this complication.

While antimicrobial resistance is caused by many factors across human, animal, and environmental domains, our findings highlight the central role of hospitals. They bring together vulnerable patients, heavy antibiotic use, and conditions that enable resistant bacteria to circulate. From a One Health perspective, hospitals are not isolated from broader systems, but are directly influenced by resistance emerging in the community, agriculture, and the environment [46,47]. By reinforcing infection prevention and control, antibiotic stewardship, and surveillance in hospitals, we also strengthen the global response to resistance across humans, animals, and the environment.

### Limitations

This study has several limitations. Its retrospective design prevented us from performing molecular microbiological analysis of resistant strains. The relatively small sample size also limits the generalizability of our results, although the cumulative antibiogram provides clinically useful insights. Future prospective studies with continuous monitoring, real-time data collection, and the integration of molecular methods will be necessary to enhance infection control and guide treatment strategies. Understanding the reality of the burden of HAIs and the difficulties in reporting is a first step in improving prevention, early identification and prompt management for healthcare-associated infections in Romania.

The importance of our study lies in the focus on a particularly vulnerable population represented by children with congenital cardiovascular anomalies, patients with cardiovascular diseases, and cardiac transplant recipients, who are significantly more susceptible to healthcare-associated infections. Factors such as frequent and prolonged hospitalizations, use of invasive devices, and long-term immunosuppression, place them at higher risk of severe morbidity and mortality.

## 4. Materials and Methods

### 4.1. Study Design and Clinical Setting

We conducted a retrospective observational study that included patients with healthcare-associated infections (HAIs) who were hospitalized at the Emergency Institute for Cardiovascular Diseases and Transplantation, Târgu Mureș, in Romania during 2023, 2024, and the first quarter of 2025. As the Institute admits only patients with congenital heart disease, cardiovascular conditions, or those requiring cardiac transplantation, the study population was limited to these high-risk groups. This study involved a descriptive analysis of all patients included, as well as an analysis to evaluate antibiotic resistance and its impact on patients diagnosed with HAIs.

### 4.2. Study Sample

Out of 13,811 admissions during the study period, 139 cases of HAIs were reported, all identified through active surveillance by the Infection Prevention and Control (IPC) Department. Among those were patients with congenital heart defects as well as recipients of cardiac transplants.

During the study period, 96 patients were diagnosed with bacterial healthcare-associated infections, for whom antimicrobial resistance patterns, cumulative antibiograms, and uni- and multivariable analyses were performed.

### 4.3. Definitions

HAIs were defined according to the Romanian national case definitions. An infection was considered hospital-acquired if symptoms developed ≥48 h after admission. HAIs also included infections occurring shortly after a previous hospitalization, such as surgical site infections (≤30 days, or ≤90 days with implants) and *Clostridioides difficile* infections within 28 days of discharge [48]. Mortality was defined as all-cause in-hospital mortality. MDR was defined as non-susceptibility to at least one agent in three or more antimicrobial categories, while XDR was defined as non-susceptibility to at least one agent in all but two or fewer antimicrobial categories [49].

Patients were eligible for inclusion if their case was reported as a HAI, provided that written consent for data storage and use had been obtained upon admission. Eligibility for the study required that clinical and microbiological data were available either in the institutional electronic medical record system or in the HAI reporting form, and that sufficient information was present to allow a follow-up of the clinical course.

Patients were excluded if essential medical data were unavailable, or if the outcome could not be evaluated due to transfer to another department or hospital.

### 4.4. Data Collection

Data were obtained from electronic medical records, the HAIs reporting form, and laboratory reports. The following variables were collected for this study: demographic information such as age and sex, department of admission, prior hospitalizations, type and site of infection, type of swab used for sample collection, microbiological results including antimicrobial susceptibility profiles, antibiotic exposure (number and classes of antibiotics received), length of hospital stay before HAI, and clinical outcomes including all-cause in-hospital mortality.

### 4.5. Ethics Approval

This study was conducted following approval from the Ethics Committee of the Institute (no. 2288/23 April 2025).

### 4.6. Microbiological Methods

Antimicrobial susceptibility testing was carried out in the hospital microbiology laboratory in accordance with the recommendations of the European Committee on Antimicrobial Susceptibility Testing (EUCAST). Susceptibility categories were assigned according to EUCAST clinical breakpoints. Results were compiled into two cumulative antibiograms, one for Gram-negative and one for Gram-positive, following standard guidelines. Those were created for the most frequent bacterial isolates obtained from patients with healthcare-associated infections during the study period. For each pathogen, resistance rates were calculated as the proportion of non-susceptible isolates to the antibiotics tested, with only the first isolate per patient included.

### 4.7. Statistical Analysis

Statistical analysis was performed using Stata statistical software, version 15.1, StataCorp, College Station, TX, USA. Odds ratios, 95% confidence intervals, and *p*-values were reported, with *p* < 0.05 considered statistically significant.

Percentages and medians were used to describe the data. An additional analysis was performed for confirmed bacterial HAI cases to assess resistance phenotypes, the impact of antimicrobial resistance, and antibiotic consumption.

In order to test associations with antimicrobial resistance, we applied the Chi^2^ test and its variant, Fisher’s exact test. Univariable logistic regression analyses were conducted to evaluate potential factors associated with death among patients with HAIs involving bacterial infections. All variables that reached statistical significance in the univariable analysis (*p* < 0.05) were subsequently included in the multivariable logistic regression model, along with age, which was incorporated *a priori* given its established clinical relevance. This approach allowed us to identify independent predictors of mortality while accounting for both statistically significant factors and a clinical risk variable.

## 5. Conclusions

This study shows that although HAIs remain underreported in Romania, the cases that are identified are often severe with a high risk of mortality. Multidrug-resistant organisms play an essential role in these outcomes, with patients frequently exposed to multiple classes of antibiotics. Strengthening surveillance systems, enforcing antibiotic stewardship, and improving infection prevention and control measures are urgently needed to reduce the burden, improve patient outcomes, and lower the costs associated with prolonged hospitalization, complex treatments, and potential legal consequences.

## Figures and Tables

**Table 1:** Univariable analysis of factors associated with mortality.

	Survived n = 55 (%)	Deceased n = 41 (%)	OR *	95%CI **	*p* Value
Year of diagnosis					
2023	23 (42)	24 (58)	Reference		
2024	25 (45)	15 (37)	0.57	0.24–1.35	0.20
2025	7 (13)	2 (5)	0.27	0.05–1.45	0.12
Sex					
Female	21 (38)	16 (39)	Reference		
Male	34 (62)	25 (61)	0.96	0.42–2.21	0.93
Age, years					
<18	25 (45)	10 (24)	0.28	0.10–0.76	0.01
18–34	2 (4)	1 (2)	0.35	0.02–4.24	0.41
35–49	4 (7)	6 (15)	1.05	0.24–4.42	0.94
50–64	10 (18)	4 (10)	0.28	0.07–1.07	0.06
>65	14 (26)	20 (49)	Base		
ICU admission					
No	28 (51)	4 (10)	Reference		
Yes	27 (49)	37 (90)	9.59	3.00–30.57	<0.001
Antimicrobial resistance					
No	34 (62)	11 (27)	Reference		
Yes	21 (38)	30 (73)	4.41	1.83–10.63	0.001
Phenotypic patterns of resistance ***					
No resistance	34 (62)	11 (27)	Reference		
ESBL	5 (9)	6 (15)	3.70	0.94–14.56	0.06
CPE	2 (4)	7 (17)	10.18	1.95–59.94	0.006
MRSA	4 (7)	1 (2)	0.77	0.07–7.66	0.82
MDR	1 (2)	0			
VRE	3 (5)	1 (2)	1.03	0.09–10.94	0.98
XDR	6 (11)	15 (37)	7.72	2.40–24.78	0.001
Number of antibiotics					
1–3	37 (67)	6 (15)	Reference		<0.001
>3	18 (33)	35 (85)	11.99	4.26–33.69	

* Odds ratio, ** confidence interval, *** ESBL—extended-spectrum beta-lactamase, CPE—carbapenemase-producing Enterobacterales, MRSA—methicillin-resistant *Staphylococcus aureus*, MDR—multidrug-resistant, VRE—vancomycin-resistant Enterococcus, XDR—extensively drug-resistant.

**Table 2:** Multivariable analysis of factors associated with mortality.

	aOR *	95%CI **	*p* Value
Age, years			
<18	0.57	0.15–2.04	0.39
18–34	0.78	0.03–19.79	0.88
35–49	4.15	0.53–32.31	0.17
50–64	0.34	0.06–1.74	0.19
>65	Reference		
ICU admission			
No	Reference		
Yes	5.89	1.39–24.91	0.016
Antimicrobial resistance			
No	Reference		
Yes	1.52	0.47–4.84	0.47
Number of antibiotics			
1–3	Reference		0.002
>3	6.79	2.00–23.03	

* Adjusted odds ratio, ** confidence interval.

## Abbreviations

The following abbreviations are used in this manuscript:

AMR	Antimicrobial Resistance
CPE	Carbapenemase-Producing Enterobacterales
DDD	Defined Daily Dose
ECDC	European Centre for Disease Prevention and Control
ESBL	Extended-Spectrum Beta-Lactamase
HAI	Healthcare-Associated Infection
ICU	Intensive Care Unit
IPC	Infection Prevention and Control
IQR	Interquartile Range
MDR	Multidrug-Resistant
MRSA	Methicillin-Resistant *Staphylococcus aureus*
VRE	Vancomycin-resistant Enterococcus
WHO	World Health Organization
XDR	Extensively Drug-Resistant
